# Dynamical states, possibilities and propagation of stress signal

**DOI:** 10.1038/srep40596

**Published:** 2017-01-20

**Authors:** Md. Zubbair Malik, Shahnawaz Ali, Soibam Shyamchand Singh, Romana Ishrat, R. K. Brojen Singh

**Affiliations:** 1 School of Computational and Integrative Sciences, Jawaharlal Nehru University, New Delhi-110067, India; 2 Centre for Interdisciplinary Research in Basic Sciences, Jamia Millia Islamia, New Delhi-110025, India

## Abstract

The stress driven dynamics of Notch-Wnt-p53 cross-talk is subjected to a few possible dynamical states governed by simple fractal rules, and allowed to decide its own fate by choosing one of these states which are contributed from long range correlation with varied fluctuations due to active molecular interaction. The topological properties of the networks corresponding to these dynamical states have hierarchical features with assortive structure. The stress signal driven by nutlin and modulated by mediator GSK3 acts as anti-apoptotic signal in this system, whereas, the stress signal driven by Axin and modulated by GSK3 behaves as anti-apoptotic for a certain range of Axin and GSK3 interaction, and beyond which the signal acts as favor-apoptotic signal. However, this stress system prefers to stay in an active dynamical state whose counterpart complex network is closest to hierarchical topology with exhibited roles of few interacting hubs. During the propagation of stress signal, the system allows the propagator pathway to inherit all possible properties of the state to the receiver pathway/pathways with slight modifications, indicating efficient information processing and democratic sharing of responsibilities in the system via cross-talk. The increase in the number of cross-talk pathways in the system favors to establish self-organization.

Natural systems are generally self-organized. Such systems, which evolve with the emergence of global coherent patterns out of local interactions^1^, are non-linear in nature^2^ with diverge equilibrium states^3^. Their spatio-temporal cross-talk with the environment and systems surrounding them try to resist any significant change in them^3^,^4^, but adapted to the changes if they find better fit new combinations provided the possibilities of adaptation subjected to a large number of stable states^5^. The responsibilities in their organization are distributed democratically among the individual components in the system^6^, favouring the coherence of the functions of individual components in the systems via interaction. To characterize such complex systems, emergent properties of both the systems and individual components constituting the systems at fundamental level are needed to be studied^7^,^8^. If one studies the dynamics of such complex system, there could be possibilities of existence of a number of perturbation driven *dynamical states* exhibiting distinct dynamical behavior in each state, which may have short or long life span, on which the system looks for its comfortable and adaptable state to exist. Since the system is generally far from equilibrium^3^ and non-linear^2^, it will hang around these dynamical states for better survival. In such studies, the systems’ properties are inherited to their respective time series data. Systematic dealing with these time series data could provide inherent properties of the complex system. Starting from the classical Mandelbrot’s mutifractal definition^9^, for a heterogeneous time series of window size *T(ϕt*), with scale factor *ϕ* and time sequence ‘*t*’, the multifractal function *M* follow the following relationships,


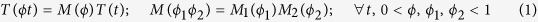


Self-similar process satisfy the simpler scaling law, *M(ϕ*) = *ϕ*^*D*^, where, *D* is self-similarity dimension^10^. Hence, time series data of a system’s dynamics generally have statistically independent multiple of such scaling behaviors exhibiting multifractal nature due to scale dependent broad probability distributions of the time series, and different long and short range correlations amplified by small and large fluctuations in the time series^11^. Then we can address fundamental issues of the system, namely, interference of noise in system dynamics, patterns of order driven by fluctuations^2^, perturbation induced change of dynamical states^12^, system’s adaptation to a change, and their implications in real biological systems.

Cellular processes are generally driven by intrinsic and extrinsic fluctuations due to random inter-molecular interaction in various cellular pathways and molecular cross-talks with the surrounding environments respectively^13^,^14^. Stress in such processes, due to external influences, abnormalities in the genetic regulations and failures of some molecular mechanisms, are propagated spatio-temporally throughout the network perturbing the pathways encountered along its path. However, the nature of stress propagation and management in the complex cellular processes is still an open question. We address these issues by modeling cross-talk of Notch-Wnt pathways in animal sometogenesis^15^,^16^,^17^,^18^ and *p*53 regulatory network^19^ via stress inducer nutilin molecule^20^,^21^,^22^. Somitogenesis, a process of periodic formation of somites in the presomitic mesoderm^23^, is regulated by the periodic expression of genes responsible in the interaction of Notch, Wnt and fibroblast growth factor pathways^18^,^24^, but control mainly by Notch and Wnt cross-talk via GSK3^25^. This Notch-Wnt cross-talk regulates their transcriptional activities and their stabilities^26^; and is responsible for variety of biological functions, for example, wing development in Drosophila^27^, T-cell development and differentiation^28^, self-renewal capacity of stem cells^29^, in a variety of carcinogenesis, such as, positive regulation in colorectal cancer cells^30^, and enhancing their expression in ovarian cancer^31^. Then p53, which is negative regulator of cellular proliferation, differentiation, and apoptotic inducer^32^, acts antagonistically to the Notch; whereas Notch can either hold back or elevates p53 activity^33^. This cross-talk of p53 and Notch-Wnt pathways by means of stress imparted in the pathways can have different regulatory mechanisms, namely, p53 activated apoptosis in T-cell Lymphocytes is suppressed by Notch signaling^34^, Notch activates p53 in neuronal progenitor cell^35^,^36^, alteration of p53 and Notch in cancer cell^37^ etc. However, these complicated cross-talks are still far from proper understanding at fundamental level. In the present work we study the interaction of p53 and Notch-Wnt pathways via stress signal which compel the system at different dynamical states, and how the system selects comfortable state out of various possibilities and adapts on it.

## Results

**Figure 1:**
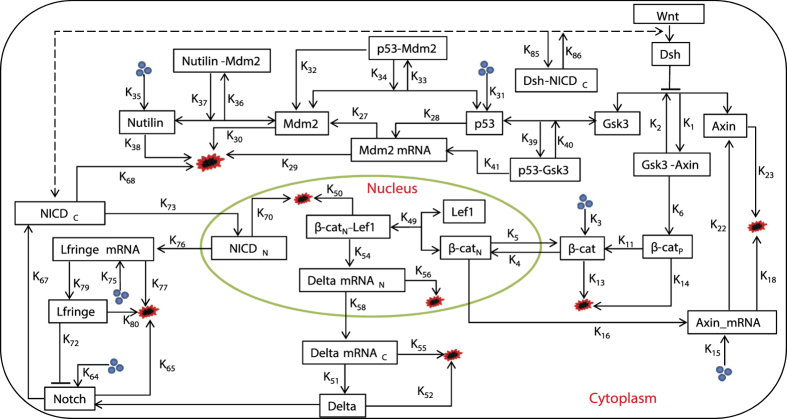
The schematic diagram of *Notch* − *Wnt* − *p*53 cross-talk model.

Regulatory biochemical network model (Fig. 1), which allows cross-talk between p53 and *Wntch* (Wnt and Notch) signaling pathways, are proposed based on some experimental reports (see Methods) to investigate complex signaling processes among them and behaviors of possible dynamical states those could correspond to certain cellular states.

### Dynamical states driven by stress signal

**Figure 2:**
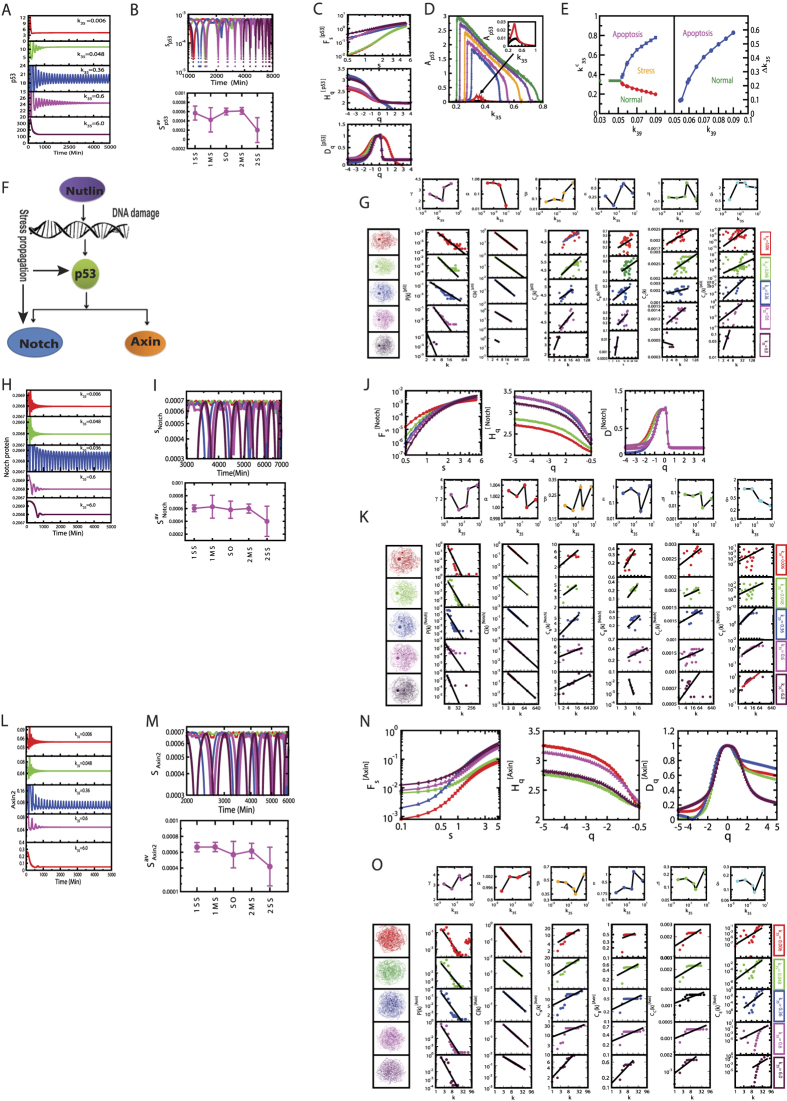
Dynamical states of stress p53 driven by nutlin, and stress propagation: (**A**) dynamical states of p53 for different *k*_35_ values, (**B**) complexity measurement characterized by calculated permutation entropy *S*_*p*53_ values of the corresponding dynamical states. (**C**) Multifractal calcualations of the dynamical states: plots of *F*_*s*_ vs *s*, 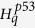 vs *q* and *D*_*q*_ vs *q*, (**D**) *A*_*p*53_ as a function of *k*_35_ for various values of *k*_39_:…., (**E**) phase diagram in the parameter space (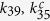), where 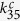 are the values of *k*_35_ cut by horizontal line, and Δ*k*_35_ is the range of *k*_35_ occupied by sustain oscillation, (**F**) schematic diagram of stress signal propagation, (**G**) topological properties of networks constructed from the time series of corresponding dynamical states: *P(k*), *C(k*), *C*_*n*_(*k*), *C*_*B*_(*k*), *C*_*C*_(*k*) and *C*_*E*_(*k*) as a function of degree *k*, (**H**) the propagated signal received by Notch and corresponding dynamical states, (**I**) permutation entropy (*H*_*Notch*_) calculation of the dynamical states, (**J**) Multifractal measures of dynamical states of Notch: plots of *F*_*s*_ vs *s*, 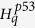 vs *q* and *D*_*q*_ vs *q*, (**K**) topological properties of the corresponding dynamical states: *P(k*), *C(k*), *C*_*n*_(*k*), *C*_*B*_(*k*), *C*_*C*_(*k*) and *C*_*E*_(*k*) as a function of degree *k*, (**L**) dynamical states of Axin due to propagated signal, and (**M**) corresponding permutation entropy (*H*_*Axin*_) values, (**N**) Multifractal calculations, (**O**) topological properties of the networks constructed from the Axin dynamical states.

The dynamical state of a system, we define here, is the dynamics of the system governed by a time series, *T(t*) which follow Mandelbrot’s Multifractal behavior^38^ throughout the time series,


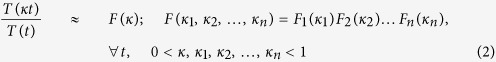


where, the fractal function *F*_*i*_(*κ*_*i*_) follows simple power law *F*_*i*_(*κ*_*i*_) ~ *κ*^*D*^ with fractal dimension *D*. Variation in concentration of nutlin trigger DNA damage^20^,^21^,^22^ (larger the nutlin concentration in the system, stronger the DNA damage), as a consequence stress signal is imparted to the system via p53 which is very sensitive to the stress signal. p53 dynamic become stabilized for low concentration of nutlin (small value of *k*_35_ ≤ 0.006), where, 
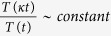
, which may correspond to nearly normal dynamical state of the system (Fig. 2A upper panel). Increase in *k*_35_ (0.006 < *k*_35_ < 0.31) drives the p53 dynamics in the mixed dynamical state, damped for a certain range of time and then stabilized, which can be expressed as, 
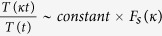
, where, *F*_*s*_(*κ*) is for certain range of time (time period and wavelength are constant, but amplitude decays). This may indicate that the system is activated first due to induced stress, and upon expended the stress the system comes back to normal. As *k*_35_ increases the range of *T*_*s*_(*κ*) also increases. For significant strong stress (0.32 < *k*_35_ < 0.52), the p53 dynamics become sustain oscillation dynamical state (range of *T*_*s*_ becomes infinite), which can be represented by, 
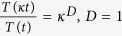
, *κ* is an integer, and *T(t*) becomes time period in this case. This dynamical state may correspond to activated state of the system where active interaction of the molecular species is involved. Further increase in *k*_35_ (0.58 < *k*_35_ < 5.1) again brings back to the mixed dynamical state. Excess stress due to nutilin (*k*_35_ > 5.3) drives the dynamics to stabilized dynamical state, which may correspond to apoptosis. Thus we could able to find five such dynamical states as listed in Table 1. The system switch to these various dynamical states in response to the stress signal.

**Figure 3:**
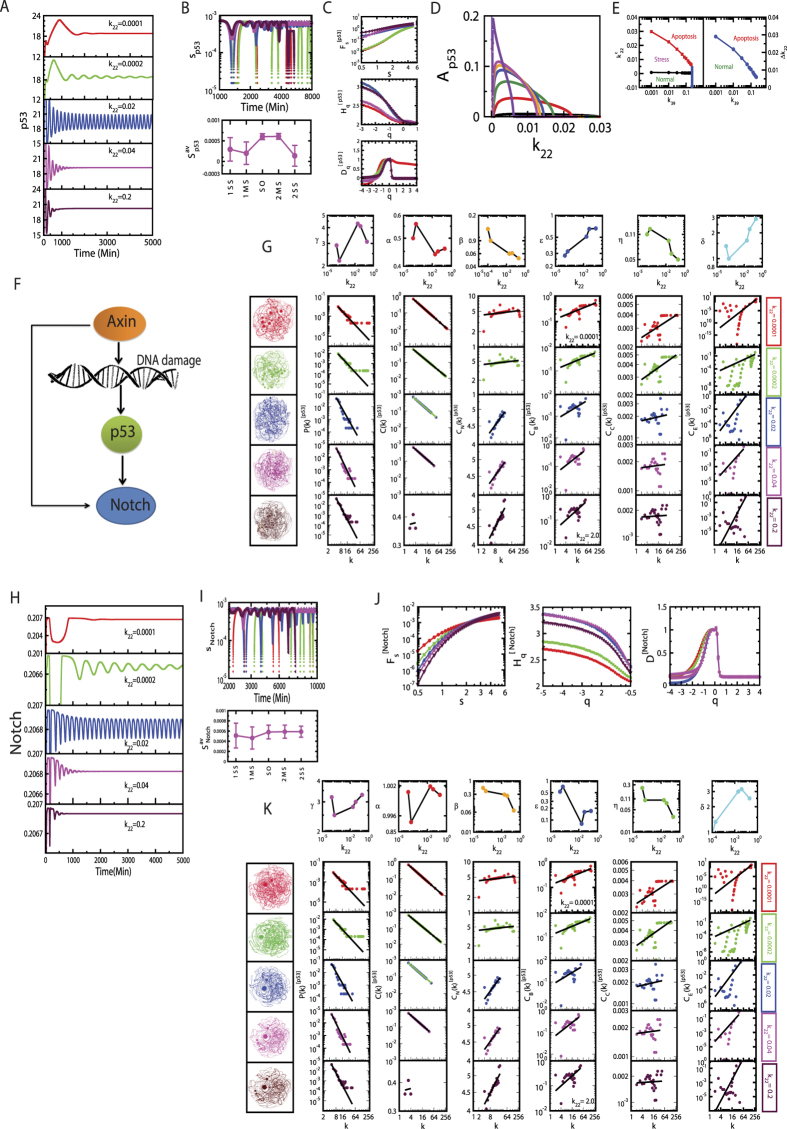
Dynamical states of stress p53 driven by Axin, and stress propagation: (**A**) dynamical states of p53 for different *k*_22_ values, (**B**) permutation entropy *S*_*p*53_ values of the corresponding dynamical states. (**C**) Multifractal calulations: plots of *F*_*s*_ vs *s*, 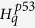 vs *q* and *D*_*q*_ vs *q*, (**D**) *A*_*p*53_ as a function of *k*_22_ for various values of *k*_39_:…., (**E**) phase diagram in the parameter space (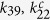), and Δ*k*_22_ is the range of *k*_22_ occupied by sustain oscillation, (**F**) schematic diagram of stress signal propagation, (**G**) topological properties of networks of corresponding dynamical states: *P(k*), *C(k*), *C*_*n*_(*k*), *C*_*B*_(*k*), *C*_*C*_(*k*) and *C*_*E*_(*k*) as a function of degree *k*, (**H**) the propagated signal receied by Notch and corresponding dynamical states, (**I**) permutation entropy (*H*_*Notch*_) calculations, (**J**) Multifractal measures, (**K**) topological properties of the corresponding dynamical states.

Another way to impart stress signal to the system could be by changing Axin concentration (changing *k*_22_) in the system which is reflected in the dynamics of p53 (Fig. 3A). Similar dynamical states, as obtained in the case of nutlin, can also be seen driven by Axin.

### Complexity in the dynamical states

The complexity of the dynamical states (Figs 2A,H,L and 3A,H) driven by stress inducer nutlin and Axin can be measured by calculating permutation entropy (*S*_*i*_, *i* → *p*53, *Notch, Axin*) of these states (*see Methods*). The calculated *S*_*i*_ magnitudes are in the order: *S*_*i*_(first stabilized state) < *S*_*i*_(first mixed state) < *S*_*i*_(sustain oscillation state) < *S*_*i*_(second mixed state) < *S*_*i*_(second stabilized state) (Figs 2B,I,M and 3B,I). The first stabilized dynamical state may correspond to normal, which is an ordered state, giving the minimum value of *S*_*i*_. Whereas, the second stabilized dynamical state corresponds to apoptotic state, which is a disorder state, providing maximum value of *S*_*i*_.

### Multifractal due to long range correlation

The stress driven p53 time series are multifractal due to short and long correlations in the time series and they might follow some probability distributions. The fluctuation variation function *F*_*s*_ (Fig. 2C upper panel) follows power law with fluctuation parameter *s* (log-log plots show approximately straight line) for all five dynamical states (Fig. 2C) with varied slopes which correspond to Hurst exponents *H*_*q*_. The *H*_*q*_ magnitudes with respect *q* for different dynamical states are as follows: *H*_*q*_(sustain oscillation) > *H*_*q*_ (second stabilized state) > *H*_*q*_(first and second mixed state)*H*_*q*_(first stabilized state). Since large *H*_*q*_ value corresponds to large fluctuations introduced in the system due to active molecular interaction driven by stress signal or internal molecular mechanisms constituted, active (sustain oscillation) dynamical state associates maximum fluctuations on an average, whereas stabilized dynamical states (both first and second) have comparatively much lower fluctuations. Since natural systems are far from equilibrium^3^, fluctuations driven systems might incorporate the fluctuations in positive way^2^ to establish their own adaptable state for better existence^5^. Further, *H*_*q*_ variation is prominent mainly in negative *q* regime as compared to the positive *q* regime, indicating multifractal nature in the time series is contributed mainly by long range correlation. This behavior is also reflected in the singularity function *F*_*α*_ as the function of *α*.

The multifractal properties of the p53 time series for various dynamical states induced by Axin show similar behavior as obtained in the case of nutlin driven p53 dynamical states (Fig. 3C). The only slight change in the nature of singularity function *F*_*α*_ which show similar behavior for all dynamical states.

### Complex stress management

The amplitude death scenario in p53 dynamics (*A*_*p*53_) as a function of *k*_35_ (Fig. 2D) could be signatures of normal (first stabilized dynamical state) and apoptotic (second stabilized dynamical state) cellular states. Monotonic is increasing and decreasing of *A*_*p*53_ as a function of *k*_35_ correspond to first and second mixed states, where removing the stress may come back to either normal or apoptotic state. Slow change in *A*_*p*53_ corresponds to sustain oscillation dynamical state, where the system is strongly activated due to active molecular interaction induced by strong stress in the system.

GSK3, the main mediator of the Notch-Wnt-p53 cross-talk, could probably regulate the transition of the system at these dynamical states by managing the stress signal. Permitting the increase in GSK3 concentration through biochemical mechanism in the system allows the system to stay in active state (sustain oscillation dynamical state) for larger range of *k*_35_ (Fig. 2D and E), trying to save the system from apoptotic phase. Therefore, GSK3 favors the system to stay in normal state for small stress signal, whereas for significantly larger stress signal, it forces the system or the system itself prefers to stay in the active state to prevent from apoptotic state (Fig. 2E). Therefore, nutlin induced stress signal along with GSK3 acts as anti-apoptotic signal in the system.

On the other hand, this stress management by GSK3 is quite different for this system when stress in it is introduced by Axin via *k*_22_ (Fig. 3D and E). Even though the dynamical states, which we found in the case of nutlin driven stress p53 dynamics, also obtained in this case, the pattern is quite opposite. In this case, increase in the concentration of mediator GSK3 (*k*_39_) the range of *k*_22_ within which sustain oscillation dynamical state can be observed becomes decreased (Fig. 3D). This increase in *k*_39_ force the available, accessible area of active state to diminish (Fig. 3E), favoring the system to go to apoptotic state. Hence, the stress signal modulated by Axin along with GSK3 acts as favored-apoptotic signal.

### Complicated self-organization

The time series of the dynamical states of stress p53 induced by nutlin (Fig. 2A) can be transformed into their respective complex networks (Fig. 2G) using visibility graph algorithm (see Methods), where, properties of the system, reflected in p53 dynamical states, can be studied using topological properties of the constructed networks. The probability of degree distribution *P(k*), clustering co-efficient *C(k*) and neighborhood connectivity *C*_*n*_(*k*) follow power law behaviour as a function of the degree *k*,


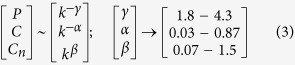


The power law behaviour in these topological properties are verified and confirmed by using the fitting technique to test power law distribution proposed by Clauset *et al*.^39^, where all statistical p-values for all data, calculated against 2500 random samplings, are found to be larger than 0.1 which is the critical limit, and goodness of fits are found to be less than and equal to 0.3. The power law behaviours of these three topological parameters are the signatures of Hierarchical features^40^ in these networks exhibiting multifractal nature in their structures. The lowest value of *γ* = 2.3 in sustain oscillation dynamical state exhibits importance of, not only system level organization of modules, but also few hubs in the network regulation. Networks corresponding to other dynamical states also follow these hierarchical properties, but the network constructed from second stabilized state (apoptotic state) lacks most of the network properties (*γ* = 4.3, *α* = 0.03, *β* = 1.5) due random distribution in the dynamics.

Centrality measurements, namely, betweenness *C*_*B*_, closeness *C*_*C*_, and eigen-vector *C*_*E*_ centralities (*see Method*), which provide the nature of information processing and to identify most influencing nodes in the network, again follow power low behaviors as a function of *k* (Fig. 2G),


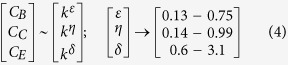


We again verify the qualification of the respective power laws to the corresponding data with the statistical technique applied above^39^. The increase in these centrality values as a function of degree (k) indicate that hubs are most influencing nodes, and take significant roles in information processing in the network. Even though the absence of these hubs do not cause network breakdown, they and their interaction among them have better responsibilities in regulating the network in each dynamical state.

The constructed networks from the dynamical states of stress p53 triggered by Axin (Fig. 3G) also show similar hierarchical properties as obtained in nutlin induced stress p53 networks. The calculated values of exponents of power law topological parameters are,


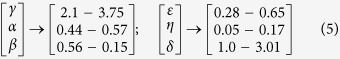


The first set of parameter values in (5) shows significant roles of hubs after sustain oscillation dynamical state. The second set of parameter shows weaker information processing as compared to nutlin induced p53 case.

### Propagation of stress signal

The stress signal exhibited in p53 dynamics induced either nutlin or Axin concentration variation is propagated to Notch and Wnt pathways through intermediate molecular interaction, and are showing up in the dynamical states of Notch and Axin (Fig. 2H and L), and Notch (Fig. 3H) respectively. The multifractal properties of dynamical states of Notch due to stress signal propagated from p53 either induced by nutlin (Fig. 2J and N), or Axin (Fig. 3J) show very similar nature as compared to that of p53 dynamical states (Fig. 2C). Further, topological parameters of the networks corresponding to dynamical states of stress receivers Notch and Wnt (due to stress inducer nutlin) (Fig. 2K and O); and Notch (due to stress inducer Axin) (Fig. 3K) exhibit similar properties as compared to the properties of the networks corresponding to dynamical states of stress propagator p53. This closely similar properties of the stress signal propagators and receivers reveals the inheritance of fundamental properties of the stress signal from propagators to receivers. Hence, the responsibilities, beneficials and injuries due to stress signal are approximately equally distributed among the important candidate proteins and/or genes in the interacting pathways. However, depending on the nature of stress signal and magnitude, and stress receivers, the inherited properties are modified according to their needs and comfortability.

Since the response of the stress propagated among the cross-talking pathways are received excellently via various mediators, the responsibilities of protecting the system as whole from any change due to external perturbations or failures in internal mechanisms might be distributed approximately equally among these pathways.

### Signature of assortivity

Hierarchically organized networks generally qualify most of the features of self-organization, namely, fractal behaviours of topological parameters, system level organization of modules and absence of central control mechanism (removal of hubs do not cause network breakdown)^41^,^42^. However, neighborhood connectivity in these networks constructed from the dynamical states of stress p53 driven by either nutlin or Axin follows, *C*_*n*_(*k*) ~ *k*^*β*^, which is power law of positive exponent (Figs 2G and 3G). The fractal behaviour is the signature of importance of few hubs and their interaction exhibiting assortivity nature of the network topology^43^,^44^. Even though the few hubs do not have the capability of full control of the network, their significantly strong cross-talks can have the possibilities of regulating the network up to some extent. In these time series of dynamical states, there could be few hubs corresponding to few time states which are responsible for signal processing and management.

The propagation of this stress signal from the propagator p53 pathway to the receivers Notch and Wnt pathways associates the assortivity characteristics. This indicates that in the time series corresponding to dynamical states of the system, interaction of few hubs (formation of the rich-club) is probably essential for efficient signal propagation and stress management.

### Active state is preferred state

The active dynamical state (sustain oscillation state) generally has maximum *H*_*q*_ values (Figs 2C,J,N and 3C,J middle pannels) due to large fluctuations than the other dynamical states. Since the system is perturbed with stress signal, the fluctuations coming from active molecular interaction due to stress signal becomes optimal, which could probably be utilized by the system in a constructive way^2^, to establish simple fractal law 
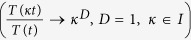
, where the system can stay comfortably with this possibility. The network, corresponding to this dynamical state, exhibit topological properties closest to hierarchical network properties^40^,^43^,


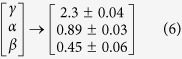


However, the noise associated with this dynamical state is far smaller than the noise at second mixed and stabilized (apoptotic) states, as evident from permutation entropy *S*_*p*53_ calculations.

### Cross-talks favor to establish self-organization

**Figure 4:**
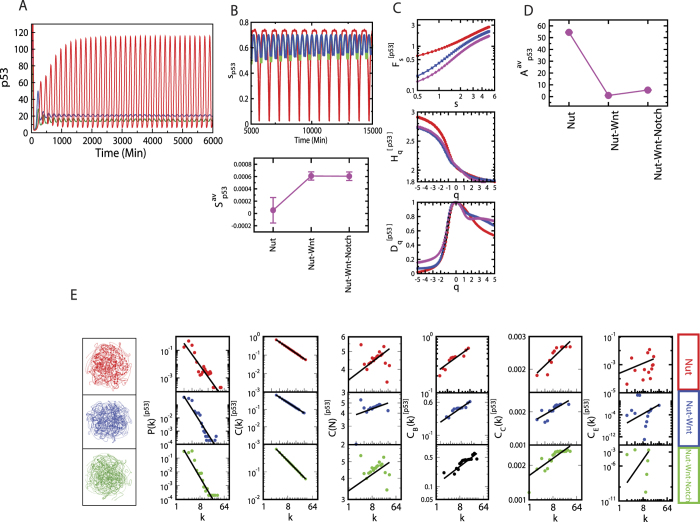
Cross-talk of pathways and properties. (**A**) Dynamics of stress p53 triggered by nutlin (red), nutlin plus Wnt (blue), and nutlin plus Wnt plus Notch (maroon), (**B**) permutation entropy measures of these cross-talks, (**C**) corresponding multifractal measures: *F*_*s*_ vs *s, H*_*q*_ vs *q*, and *D*_*q*_ vs *q* plots, (**D**) *A*_*p*53_ corresponding to the cross-talks, (**E**) topological properties of the corresponding networks: *P(k*), *C(k*), *C*_*n*_(*k*), *C*_*B*_(*k*), *C*_*C*_(*k*) and *C*_*E*_(*k*) as a function of degree *k*.

Stress p53 dynamics driven by nutlin exhibits optimized active dynamical state as number of cross-talks of pathways are increased (Fig. 4A–E). *S*_*i*_ for p53 in the p53 pathway (direct stress is introduced via nutlin) is maximum, then the *S*_*i*_ is pulled down drastically as it just cross-talks to Wnt pathway. When Notch-Wnt-p53 cross-talk is established *S*_*i*_ is slightly increased, may be trying to optimize the fluctuations imparted by active molecular interaction due to stress in the system. Calculated *H*_*q*_ value is minimized as number of cross-talks of pathways is increased (Fig. 4C), but amplitude *A*_*p*53_ is optimized (Fig. 4D).

The constructed networks from these p53 dynamics corresponding to different combinations of cross-talks of the pathways (Fig. 4E) show that the topological properties of the network corresponding to Notch-Wnt-p53 interaction exhibits better organized and closer to hierarchical features,


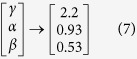


The assortivity property, indicating the importance of hubs in the network, is still one of the important properties of the network. The important responsibilities of these hubs in information processing in the network are reflected and become clear in the fractal nature of the centrality measurements.

## Discussion and Conclusion

Natural systems always try to be self-organized, subjected to a number of possibilities to change and corresponding states which are far from equilibrium, try to adapt to the preferred changes beneficial to their own organization for better and comfortable survival. During this struggle, the system tries to optimize any change (such as fluctuations due to stress in the system) in a constructive manner, utilize and distribute it, and look for better stability. If the system cannot able to face the change, it will break down.

Stress induced in Notch-Wnt-p53 cross-talk by stress inducing molecules (nutlin and Axin in our case), which are exhibited in stress p53 dynamics, drives the system at various dynamical states defined by different fractal laws, and the system switch to these dynamical states depending on the amount of stress induced. This stress system prefers to stay in an active dynamical state which has simple fractal rule subjected to the optimal fluctuations available due to active molecular interaction driven by stress. However, the system still associates a group of few hubs (assortive topology), but not in dependent manner (absence of these hubs do not cause system’s breakdown), for better signal processing and system regulation. Then this stress signal is propagated throughout the pathways, and found to inherit all the properties of the propagator pathway to the receiver pathways may be with slight modifications in them. This excellent co-ordination in cross-talk helps the system to save it from one directional apoptosis (once the system falls in this phase, it can never come back to normal situation) by regulating available active molecular interaction. This regulating mechanism could be different depending on the type of stress induced in the system (nutlin and Axin in our case).

The dynamical states obtained in our simple model are simple governed by simple fractal rules with limited possibilities. Multifractal studies show that these states are triggered by long range correlations with varied fluctuations. However, natural complex systems can have complex dynamical states defined by complex fractal rules allowing large number of possibilities in it.

The increase in the number of cross-talk pathways allows the system to optimize the imparted fluctuations, and therefore favours to establish self-organization in the whole system. This cross-talk regulates various biological functions out of which is regulating apoptosis. In the case of nutlin induced stress system, nutlin along with cross-talk mediator GSK3 acts as anti-apoptotic signal. Whereas, Axin induced stress along with GSK3 acts in two fold, first as an anti-apoptotic within a certain range of their interaction range, second favour-apoptotic signal beyond that regime. Since this Notch-Wnt-p53 cross-talk is involved in many important cellular processes in normal and cancerous cells, one needs to investigate these cellular activities with a number of open questions to understand how they work at a fundamental level.

## Methods

### Coupled Wntch (i.e. Wnt/Notch) Signaling in development

The notion Wntch signaling was introduced by Martinez Arias in 2008, for the elements of integrated system controlling cell fate i.e. Wnt and Notch^45^. The most important part of multicellular organisms is development which requires the proliferation of cell that decides its fate^46^. This decision for fate determination converge on the enhancement of specific genes that create a combination of transcription factors that determine the state and behaviour of the cell^47^. According to this analysis obtained from the study of *Dorsophila* genetics it can be proposed that Wnt is involved in regulating the probability of cell fate adoption^48^. The current understanding of Wnt signalling gives two striking features as reviewed by Martinez Arias and Hayward 2006, first is that many elements of this pathway take part in many other signaling processes, and second, is the ability of Wnt signaling to co-operate with other transcription factors and modulate their effect in other signaling pathways^48^. Thus, it acts as a noise filter, i.e making fluctuation in gene expression less strong^49^. However, there is one pathway that is consistent with Wnt, which is generally known as Notch signaling pathway^48^. Since Notch is involved in lateral inhibition, it together with Wnt plays crucial role in patterning the Notch inhibitory process^47^. Since, the development of wings in *Dorsophila* was first established by Couso and Martinez Arias in 1994, it also gave us a strong proof of interaction of notch and Wnt with strong sensitivity^50^. These evidences indicate that the elements of Notch and Wnt form a closed pathway for information processing, where Notch is available for NICD and Wnt regulate the Notch traffic using the *β*-catenin and activity of GSK3 in specific cells. Thus, Wntch signaling gives a clear picture of transition of states in cell during development^51^.

### Notch-Wnt-p53 cross-talk biochemical network

Notch-Wnt cross-talk is a well-studied model^16^,^17^,^18^,^25^,^52^,^53^ which regulates variety of biological functions in normal and cancer cells, from lower to higher level organisms. On the other hand, p53 is very sensitive in responding stress in the system, may be via DNA damage or due to other molecular mechanisms, and drives the cell at various cellular states and lets the cells to decide their fate of survival^19^,^54^. The cross-talk between Notch-Wnt and p53 pathways are particularly important to understand, (i) how does p53 interfere Notch-Wnt interaction, and vice versa, (ii) nature of stress signal propagation in interacting pathways, and (iii) regulation of apoptosis. We present here Notch-Wnt-p53 regulatory biochemical network (Fig. 1) by incorporating simplified versions of the above models and other experimental findings.

### Model Description

Wnt signaling pathway allows Wnt to interact with Dsh (Desheilved protein) to inhibit the formation of ubicutinaceous complex between Gsk3*β* and Axin2, which de-phosphorylate *β*-catenin (*k*_6_) to enter the nucleus (with a rate *k*_4_) and initiates the expression of Axin2-mRNA (*k*_16_). Then Axin2-mRNA is transported into the cytoplasm as part of the negative feedback loop between Wnt and Axin2. Free Gsk3*β* available in the system will allow to communicate Wnt with p53 by competing with the binding of p53 and Mdm2, with the formation of complex Gsk3*β* with p53 (*k*_39_). The process then activates the transcription of *Mdm*2-mRNA (*k*_41_), and it is translated (*k*_27_) and translocated to the cytoplasm where it binds to unbound p53 to degrade (*k*_33_). On the other hand, *β*-*catenin* present in the nucleus binds to Lef1 (*k*_49_) to increase the expression of mRNA of Delta gene (*k*_54_), followed by translation of Delta (*k*_51_) and translocated to the cytoplasm (*k*_58_). Now, this Delta protein interacts with *Notch*–*NICD* binary complex present on the cell membrane^55^ to initiate the release of NICD to form ternary complex (*k*_67_) and facilitating its transport to the nucleus (*k*_73_). The release of NICD in the cytoplasm is due to the ligand Delta that performs two activities cis-inhibition (intracellular i.e degradation of Notch) and trans-activation (intercellular) of Notch^53^,^55^. In our case cis-inhibition and trans-activation goes simultaneously within the cell, where cis-inhibition degrades Notch with rate constant *k*_65_ and trans-activation of Notch releases NICD in Cytoplasm (see Supplementary Fig. 1) followed by the nuclear transportation^55^. Further, NICD in the nucleus triggers the expression of Lfringe (mRNA) (*k*_76_)^56^ followed by its translation (*k*_79_). After translation, Lfringe goes to bind to its site on Notch followed by the inhibition of formation Notch delta complex to inhibit its own (Lfringe) transcription (*k*_76_) exhibiting negative feedback loop between Notch and Lfringe^57^. Lfringe does not prevent the binding of DSL ligands to Notch, while it potentiates both via Notch^58^. On the other hand, Lfringe decreases the binding of Notch and Delta by modifying (*O*-linked fucose glycosylation) the extracellular region of Notch2^59^. However, Wnt directly inhibits the formation of Lfringe by direct transfer of information via interaction of Dsh and NICD present in cytoplasm (*k*_86_). The periodic expression of Lfringe is essential for somite formation^56^. Notch pathway interacts with Wnt via a mediator molecule Dsh, that bind to the cytoplasmic form of NICD (NICDc) (*k*_85_). This Dsh inhibits the formation of the destruction complex (formed by the association of GSK3*β*, Axin and *β*-catenin). GSK3*β* from this complex binds to p53 in the presence of Mdm2 forming binary complex p53-GSK3*β (k*_39_). This binary complex activates the transcription of Mdm2 with a rate constant of 0.042 *min*^−1^ (*k*_41_), increasing the concentration of Mdm2 inside the cell. This hike in the Mdm2 decreases the p53 concentration (*k*_33_). The bound p53 is restored by Nutilin (*k*_36_), a small molecule that compete to the binding site of p53 on Mdm2. On the other hand activated *β*-catenin is translocated into the nucleus (*k*_4_) and bind with the Lef1 (*k*_49_) to activate the transcription of Delta in the nucleus (*Delta* mRNA). This *Delta* mRNA is translated in cytoplasm to the protein that go to Notch to promote the cleavage of NICD (*k*_51_). The interaction of Notch-Wnt-p53 pathways allows to regulate apoptosis as well as stress propagation.

### Mathematical framework of the network

The Notch-Wnt-p53 regulatory network model (Fig. 1) is defined by *N* = 28 molecular species (Table 2) corresponding to the reaction network description provided in Table 3. The state of the system at any instant of time ‘*t*’ is given by the state vector, 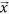 (*t*) = (*x*_1_, *x*_2_, …, *x*_*N*_)^*T*^, where, *N* = 28 and ‘*T*’ is the transpose of the vector. By considering feedback mechanism of in *p*53, Wnt and Notch oscillators and coupling reaction channels of the two oscillators, we could able to reach the following coupled ordinary differential equations (ODE),


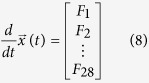


where, the functions in the equation (8) {*F*_*i*_(*x*_1_, *x*_2_, …, *x*_*N*_)}, *i* = 1, 2, …, 28 are given by,


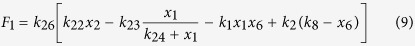



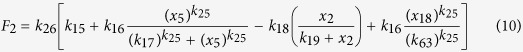



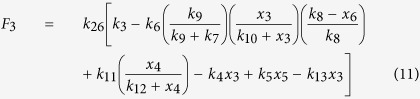



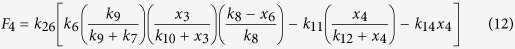



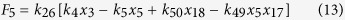



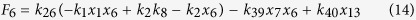



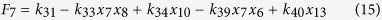



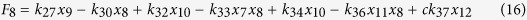



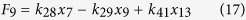



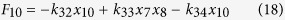



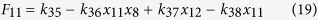



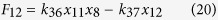



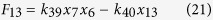



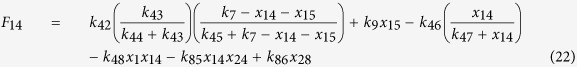



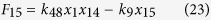



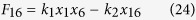



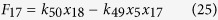



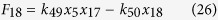



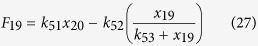



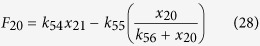



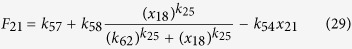



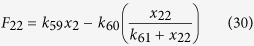



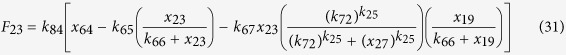



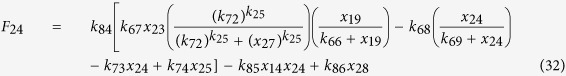



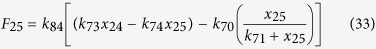



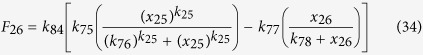



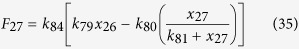



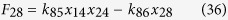


We used standard Runge-Kutta method (order 4) of numerical integration to simulate equation (8) to find the solution of the variables listed in Table 2 for the parameter values given in Table 3. We then analyzed the constructed mathematical model to get possible approximate analytical solutions of the variables (slow variables) using quasi-steady state approximation.

### Multifractal DFA approach

Fractal properties in non-stationary time series, and associated important correlations can studied using Multifractal detrended fluctuation analysis (MF-DFA)^60^. Important fractal parameters which characterize the time series, namely, Hurst exponent (*H*), generalized dimension (*D*) etc can be calculated numerically using a method adopted by Kantelhardt *et al*.^11^ as summarized below. First, the time series signal {*x*_*j*_} of length *N* is taken as random walk, and can be represented by the profile, 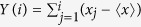, where, 〈*x*〉 is the mean value of the signal, and *i* = 1, 2, …, *N*. Second, the profile *Y(i*) is now divided into 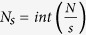 equal non-overlapping equal segments of size *s*. To take into account all data points, 2*N*_*s*_ segments are considered by counting starting from both ends of the data. Third, the following variance is determined,


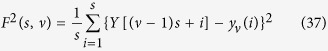


where, *ν* = *N*_*s*_ + 1, …, 2*N*_*s*_, and *y*_*ν*_(*i*) is the fitting polynomial in segment *ν*. Fourth, the *q*^*th*^ order fluctuation function is estimated by averaging over all segments,


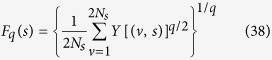


Fifth, the scaling behavior of the function *F*_*q*_(*s*) is represented by,


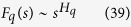


where, *H*_*q*_ is the generalized Hurst exponent, which represents the measure of self-similarity and correlation properties of the signal. Then *H*_*q*_ is related to the classical scaling exponent *τ(q*),


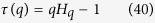


and from the definition of Holder exponent, 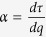, the singularity function *f(α*)^60^ is given by,


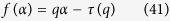


Then, generalized fractal dimension of the signal is measured by,


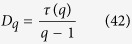


Now, *D*_0_, for *q* = 0, is the fractal or Hausdorff dimension, *D*_1_ is information dimension and *D*_2_ represents correlation dimension^60^. Multifractal signature in the time series can be observed in the system if there exists significant dependence of *H*_*q*_ on *q* in the time series due to the different scaling nature of small and large fluctuations^11^. Positive dependence of *H*_*q*_ on *q* indicates short range correlations in the time series, whereas negative dependence of *H*_*q*_ on *q* exhibits long range correlations in the time series. Further, in multifractal time series, small and large fluctuations are characterized by large and small values of *H*_*q*_.

### Visibility graph of time series

This technique maps a time series to a network^61^, where each observation in time series is translated to a node and an edge between any two nodes is introduced when the following visibility condition is satisfied i.e. two nodes corresponding to observations *x(t*_*a*_) and *x(t*_*b*_) are connected if all intermediate states *x(t*_*c*_) with *t*_*a*_ < *t*_*c*_ < *t*_*b*_ satisfy,


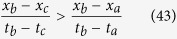


These networks are undirected due to symmetry in visibility condition. Since the properties of the time series are inherited to the corresponding network, the studies of this network provide useful information which can’t be observed in traditional time series data.

### Topological properties of networks

The following topological properties of the networks are studied to study the important behavior of the networks.

#### Degree distribution

The degree *k* of a node indicates the number of links the node connects with other nodes in the network. Consider a network defined by a graph *G* = (*N, E*), where *N* and *E* are number of nodes and edges respectively. The probability of degree distribution (*P(k*)) of the network is the probability that any chosen node will have a degree *k*, which is given by,


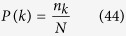


where, *n*_*k*_ is the number of nodes having degree *k. P(k*) in random and small-world networks follow Poisson distribution, whereas, it obeys power law *P(k*) ~ *k*^−*γ*^ in scale-free and hierarchical networks depending on the value of *γ* which indicates the importance of hubs or modules in the network^62^.

#### Clustering co-efficient

Clustering co-efficient of a network characterize how strongly a node’s neighborhood nodes are interconnected. For an undirected network, clustering co-efficient (*C(k*_*i*_)) of ith node is the ratio of number of its nearest neighborhood edges to the total possible number of edges of degree *k*_*i*_, and can be calculated by,


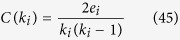


where, *e*_*i*_ is the number of connected pairs of nearest-neighbor of ith node, and *k*_*i*_ is its degree. *C(k*) in scale free networks is independent of *k*, whereas in hierarchical network it follows a power law, *C(k*) ~ *k*^−*α*^, with *α* ~ 1.

#### Neighborhood connectivity

Neighborhood connectivity of a node of a network is a measure of the average connectivities of the nearest neighbors of the node in the network^63^, and is given by,


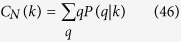


where, *P(q*|*k*) is conditional probability that a link belonging to a node with connectivity *k* points to a node with connectivity *q*. The power law nature of *C*_*N*_(*k*), *C*_*N*_(*k*) ~ *k*^−*β*^ is a signature of hierarchical topology in the network^43^. However, the positive power dependence of *C*_*N*_(*k*) could be an indicator of assortivity in the network topology^44^.

#### Betweenness centrality

Betweenness centrality of a node characterize the ability to (i) extract benefits from information flows in the network^64^, and (ii) extent to which the node has control over the other nodes in the network through communication^65^,^66^. If *d*_*ij*_(*v*) indicates the number of geodesic paths from node *i* to node *j* passing through node *v*, and *d*_*ij*_ represents number of geodesic paths from node *i* to *j*, then betweenness centrality (*C*_*B*_(*v*)) of a node *v* can be measured by,


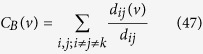


If *M* denotes the number of node pairs excluding *v*, then normalized betweenness centrality is given by, 
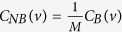
.

#### Closeness centrality

Closeness centrality (*C*_*C*_) estimates how fast information is spread from a node to other nodes reachable from it in the network^67^. *C*_*C*_ of a node *i* is the harmonic mean of geodesic distance between the node and all other nodes connected to it in the network,


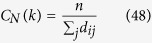


where, *d*_*ij*_ is geodesic path length between nodes *i* and *j*, and *n* is the total number of nodes in the network connected to node *i*.

#### Eigenvector centrality

Eigenvector centrality of a node *i (C*_*E*_(*i*)) in a network is proportional to the sum of *i*′s neighbor centralities^68^, and it is measured by,


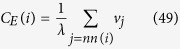


where, *nn(i*) indicates nearest neighbors of node *i* in the network. *λ* is the eigenvalue of the eigenvector *v*_*i*_ given by, *Av*_*i*_ = *λv*_*i*_, where, *A* is the adjacency matrix of the network. The principal eigenvector of *A*, which corresponds to maximum eigenvalue *λ*_*max*_, is taken to have positive eigenvector centrality score^69^. Since node’s eigenvector centrality function smoothly varies over the network and depends on its neighbors, node with high eigenvector centrality is embedded in the locality of nodes of high eigenvector centralities, and chance of having isolated nodes in and around the locality is very low^68^. Hence, eigenvector centrality can be used as an indicator of node’s spreading power in the network.

### Algorithm for calculating permutation entropy

Important information contained in a time series can be measured by calculating permutation entropy of the time series^70^,^71^. Permutation entropy *H* of a time series of a dynamical variable *x(t*) of a system can be calculated as follows. The time series *x(t*) can be mapped onto a symbolic sequence of length *N: x(t*) = {*x*_1_, *x*_2_, …, *x*_*N*_}. This sequence is then partitioned into *M* number of short sequences of equal length *U* i.e. *x(t*) = {*w*_1_, *w*_2_, …, *w*_*M*_}, where *i*th window is given by *w*_*i*_ = {*x*_*i*+1_, *x*_*i*+2_, …, *x*_*i*+*U*_}. This window is allowed to slide along *x(t*) with maximum overlap. Permutation entropy of a window *w*_*i*_ can be calculated by defining a short sequence of embedded dimension *r, S*_*i*_ = {*x*_*i*+1_, *x*_*i*+2_, …, *x*_*i*+*r*_} in *r*-dimensional space, finding all possible inequalities of dimension *r* and mapping the inequalities along the ascendingly arranged elements of *w*_*i*_ to find the probabilities of occurrence of each inequality in *w*_*i*_. Since *q* out of *r*! permutations are distinct, one can define a normalized permutation entropy as 
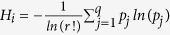
, where 0 ≤ *H*_*i*_(*r*) ≤ 1. The mapped permutation entropy spectrum of time series *x(t*) is represented by *H* = {*H*_1_, *H*_2_, …, *H*_*M*_}. In self-organized state one has *H* → 0.

## Additional Information

**How to cite this article**: Malik, M. Z. *et al*. Dynamical states, possibilities and propagation of stress signal. *Sci. Rep.* **7**, 40596; doi: 10.1038/srep40596 (2017).

**Publisher's note:** Springer Nature remains neutral with regard to jurisdictional claims in published maps and institutional affiliations.

## Supplementary Material

Supplementary Information

## Figures and Tables

**Table 1:** Different Dynamical state.

S.No.	Dynamical state	Description	Reference
1.	First steady state	May correspond to normal cell.	72, 73, 74, 75
2.	First damped oscillation state	Stress (weak) induced cell that could able to repair back the changes in the cell and may come back to the normal condition.	72, 73, 74, 75
3.	Sustain oscillation state	Active state of the cell where active interaction of molecules are going on in the cell.	72, 73, 74, 75
4.	Second damped oscillation state	Damped oscillation state where the strong stress in p53 due to stress inducer nutlin and axin may become toxic to the system. In this state cell is associated with large stress, which cannot be repaired back and may probably go to apoptosis after some time.	see Supplementary information
5.	Second steady state	Apoptotic state where repairment of pathways cannot force cell to become normal in excess stress condition, thus cell chooses to destroy itself.	72, 73, 74, 75

**Table 2:** List of molecular species and their initial concentration.

S.No.	Species Name	Description	Notation	Initial Concentration (nM)
1.	Axin2	Initial Concentration *Axin*2 protein	*x*_1_	10.1
2.	*Axin*2 mRNA	Initial Concentration *Axin*2 mRNA	*x*_2_	0.02
3.	*β*-catenin	UnPhosphorylated *β*-catenin	*x*_3_	0.1
4.	*β*-catenin_*P*_	Phosphorylated *β*-catenin	*x*_4_	0.1
5.	*β*-catenin_*N*_	Nuclear *β*-catenin	*x*_5_	0.01
6.	Gsk3	*Gsk*3 protein	*x*_6_	5.0
7.	p53	unbound *p*53 protein	*x*_7_	10.0
8.	Mdm2	Unbound *Mdm*2 protein	*x*_8_	10.0
9.	*Mdm*2 mRNA	*Mdm*2 Messsenger mRNA	*x*_9_	0.0
10.	p53-Mdm2	*Mdm*2 and *p*53 complex	*x*_10_	95.0
11.	Nutlin	Unbound Nutlin	*x*_11_	5.0
12.	Nutlin-Mdm2	Nutlin *Mdm*2 complex	*x*_12_	0.0
13.	p53-Gsk3	*p*53 and *Gsk*3 complex	*x*_13_	0.0
14.	Dsh-activated	Activated Dsh protein	*x*_14_	0.5
15.	Dsh-Axin	The complex of Dsh and Axin2	*x*_15_	2.0
16.	Gsk3-Axin2	The complex of *Gsk*3 and *Axin*2	*x*_16_	2.0
17.	Lef1	The Lef1 protein in nucleus	*x*_17_	0.1
18.	*β*-cat_*N*_-Lef1	The complex of *β*-catenin and Lef1 protein	*x*_18_	0.01
19.	Delta	The Delta protein	*x*_19_	1.68
20.	*Delta* mRNA_*C*_	The mRNA of Delta in the cytoplasm	*x*_20_	0.1
21.	*Delta* mRNA_*N*_	The mRNA of Delta in the nucleus	*x*_21_	0.1
22.	*Axin*2 mRNA_*C*_	The mRNA of Axin2 in the cytoplasm	*x*_22_	0.1
23.	Notch	The Notch protein	*x*_23_	0.5
24.	NICD_*C*_	Active Notch protein in the cytoplasm	*x*_24_	0.2
25.	NICD_*N*_	Active Notch protein in the nucleus	*x*_25_	0.0
26.	*Lfring* mRNA	The mRNA of Lunatic fringe protein	*x*_26_	0.01
27.	Lfring	The Lunatic fringe protein	*x*_27_	0.1
28.	Dsh-NICD_*C*_	The complex of Dsh and active Notch protein in cytoplasm	*x*_28_	0.1

**Table 3:** List of parameters.

S.No.	Notation	Description	Values	Reference
1.	*K*_1_	Rate constant for binding of *Gsk*3 to *Axin*2	0.23 *nMmin*^−1^	16
2.	*K*_2_	Rate constant for dissociation of *Gsk*3-*Axin*2	0.1 *min*^−1^	16
3.	*K*_3_	Rate constant of *β*-catenin synthesis	0.087 *nMmin*^−1^	16
4.	*K*_4_	Rate constant of *β*-catenin entry into the nucleus	0.7 *min*^−1^	16
5.	*K*_5_	Rate constant of *β*-catenin exit from the nucleus	1.5 *min*^−1^	16
6.	*K*_6_	Rate constant of phosphorylation of *β*-catenin by the *Gsk*3	5.08 *nMmin*^−1^	16
7.	*K*_7_	Concentration of Dishevelled(Dsh)protein	2.0 *nM*	16
8.	*K*_8_	Total *Gsk*3 Concentration	3.0 *nM*	16
9.	*K*_9_	Rate constant of inhibition by Dsh for*β*-catenin_*P*_ by the *Axin*2-*Gsk*3 complex	0.5 *nM*	16
10.	*K*_10_	Michaelis rate constant for *β*-catenin_*p*_ by the *Axin*2-*Gsk*3 complex	0.28 *nM*	16
11.	*K*_11_	Maximum rate constant of dephosphorylation of *β*-catenin	1.0 *nMmin*^−1^	16
12.	*K*_12_	Maximum rate constant for *β*-catenin phosphoration	0.003 *nM*	16
13.	*K*_13_	Rate constant for degradation of unphosphorylated *β*-catenin	0	16
14.	*K*_14_	Rate constant for degradation of phosphorylated *β*-catenin	7.062 *min*^−1^	16
15.	*K*_15_	Rate constant of transcription of the *Axin*2 gene	0.06 *nMmin*^−1^	16
16.	*K*_16_	Rate constant of transcription of the *Axin*2 gene induced by nuclear *β*-catenin	1.64 *nMmin*^−1^	16
17.	*K*_17_	Rate constant for induction by nuclear *β*-catenin of *Axin*2 gene trascription	0.7 *nM*	16
18.	*K*_18_	Maximum rate constant of degradation of *Axin*2 mRNA	0.8 *nMmin*^−1^	16
19.	*K*_19_	Rate constant for degradation of *Axin*2 mRNA	0.48 *nM*	16
20.	*K*_20_	Rate constant of transcription of *Axin*2 gene induced by transcription factor	0.5 *nMmin*^−1^	16
21.	*K*_21_	Rate constant for induction by transcription factor of *Axin*2 gene transcription	0.05 *nM*	16
22.	*K*_22_	Rate constant of synthesis of *Axin*2 protein	0.02 *min*^−1^	16
23.	*K*_23_	Maximum rate constant of degradation of *Axin*2 protein	0.6 *nMmin*^−1^	16
24.	*K*_24_	Michaelis rate constant for degradation of *Axin*2 protein	0.63 *nM*	16
25.	*K*_25_	Hill coefficient	2.0	16
26.	*k*_26_	Scaling factor for Wnt oscillator	1.5	16
27.	*k*_27_	Rate constant of *Mdm*2 synthesis	0.297 *min*^−1^	19
28.	*k*_28_	Rate constant of *Mdm*2 transcripton	0.006 *min*^−1^	19
29.	*k*_29_	Rate constant of *Mdm*2 mRNA degradation	0.006 *min*^−1^	19
30.	*k*_30_	Rate constant *Mdm*2 degradation	0.2598 *min*^−1^	19
31.	*k*_31_	Rate constant of p53 synthesis	4.68 *min*^−1^	19
32.	*k*_32_	Rate constant of p53 degradation	0.0495 *min*^−1^	19
33.	*k*_33_	Rate constant of p53-Mdm2 binding	0.693 *min*^−1^	19
34.	*k*_34_	Rate constant of *p*53-*Mdm*2 dissociation	0.00693 *min*^−1^	19
35.	*k*_35_	Rate constant of Nutilin formation	0.001 *min*^−1^	Estimated
36.	*k*_36_	Rate constant of Nutilin-*Mdm*2 formation	0.012 *min*^−1^	Estimated
37.	*k*_37_	Rate constant of Nutilin-*Mdm*2 dissociation	0.03 *min*^−1^	Estimated
38.	*k*_38_	Rate constant of Nutilin degradation	0.06 *min*^−1^	Estimated
39.	*k*_39_	Rate constant of p53-Gsk3 complex formation	0.04 *min*^−1^	76
40.	*k*_40_	Rate constant of p53-Gsk3 complex dissociation	0.12 *min*^−1^	76
41.	*k*_41_	Rate constant of *Mdm*2 mRNA synthesis	0.042 *min*^−1^	76
42.	*k*_42_	Rate constant of activation of Dsh by Wnt	5.0 *min*^−1^	25
43.	*k*_43_	Wnt protein concentration	5.0 *min*^−1^	25
44.	*k*_44_	Michaelis rate constant for activation of Dsh by Wnt	1.5 *min*^−1^	25
45.	*k*_45_	Michaelis rate constant for activation of Dsh	0.95 *min*^−1^	25
46.	*k*_46_	Rate constant of activation of inactivation of Dsh	1.0 *min*^−1^	25
47.	*k*_47_	Michaelis rate constant for inactivation of Dsh	0.647 *min*^−1^	25
48.	*k*_48_	Bimolecular rate constant for complex formation of Asin2 and Dsh	0.9 *min*^−1^	25
49.	*k*_49_	Bimolecular rate constant for complex formation of *β*-catenin to Lef1 protein	0.9 *min*^−1^	25
50.	*k*_50_	Bimolecular rate constant for degradation of complex of *β*-catenin to Lef1 protein	0.9 *min*^−1^	25
51.	*k*_51_	Apparent first-order rate constant for the formation of Delta protein	0.1 *min*^−1^	25
52.	*k*_52_	Maximum rate constant of degradation of Delta protein	0.9 *min*^−1^	25
53.	*k*_53_	Michaelis rate constant for degradation of Delta protein	5.0 *min*^−1^	25
54.	*k*_54_	Maximum rate constant of transcription of Delta gene induced by the complex of *β*-catenin and Lef1 in nucleus	1.12 *min*^−1^	25
55.	*k*_55_	Maximum rate constant of degradation of mRNA of Delta protein in cytoplasm	2.0 *min*^−1^	25
56.	*k*_56_	Michaelis rate constant for degradation of mRNA of Delta protein in cytoplasm	1.0 *min*^−1^	25
57.	*k*_57_	Basal rat constant of transcription of the delta gene	1.0 *min*^−1^	25
58.	*k*_58_	Apparent first-order rate constant for mRNA of Delta protein exit from the nucleus	1.725 *min*^−1^	25
59.	*k*_59_	Apparent first-order rate constant for mRNA of Axin2 exit from the nucleus	2.0 *min*^−1^	25
60.	*k*_60_	Maximum rate constant of degradation of mRNA of Axin2 in cytoplasm	1.2 *min*^−1^	25
61.	*k*_61_	Michaelis rate constant for degradation of mRNA of Axin2 in cytoplasm	0.48 *min*^−1^	25
62.	*k*_62_	Threshold rate constant for transcription of Delta induced by the complex of *β*-catenin and Lef1 protein in nucleus	2.46 *min*^−1^	25
63.	*k*_63_	Threshold rate constant for transcription of Axin2 induced by the complex of *β*-catenin and Lef1 protein in nucleus	2.46 *min*^−1^	25
64.	*k*_64_	Maximum rate constant of formation of Notch	0.23 *min*^−1^	16
65.	*k*_65_	Maximum rate constant of degradation of Notch	2.82 *min*^−1^	16
66.	*k*_66_	Michaelis rate constant for degradation of Notch	1.4 *min*^−1^	16
67.	*k*_67_	Apparent first-order rate constant for Notch cleavage into NICD	3.45 *min*^−1^	16
68.	*k*_68_	Maximum rate constant of degradation of NICD	0.01 *min*^−1^	16
69.	*k*_69_	Michaelis rate constant for degradation of NICD	0.001 *min*^−1^	16
70.	*k*_70_	Maximum rate constant of degradation of nuclear NICD	0.01 *min*^−1^	16
71.	*k*_71_	Michaelis rate constant for degradation of nuclear NICD	0.001 *min*^−1^	16
72.	*k*_72_	Threshold rate constant for inhibition of Notch cleavage into NICD by Lunatic fringe	0.5 *min*^−1^	25
73.	*k*_73_	Apparent first-order rate constant for NICD entry into nucleus	0.1 *min*^−1^	16
74.	*k*_74_	Apparent first-order rate constant for NICD exit from the nucleus	0.1 *min*^−1^	16
75.	*k*_75_	Maximum rate constant of transcription of *Lunaticfringe* gene	3.0 *min*^−1^	16
76.	*k*_76_	Threshold rate constant for activation of *Lunaticfringe* gene transcription by nuclear NICD	0.05 *min*^−1^	16
77.	*k*_77_	Maximum rate constant of degradation of *Lunaticfringe* mRNA	1.92 *min*^−1^	16
78.	*k*_78_	Michaelis rate constant of degradation of *Lunaticfringe* mRNA	0.768 *min*^−1^	16
79.	*k*_79_	Apparent first-order rate constant for *Lunatic fringe* protein synthesis	0.3 *min*^−1^	16
80.	*k*_80_	Maximum rate constant of *Lunatic fringe* protein degradation	0.39 *min*^−1^	16
81.	*k*_81_	Michaelis rate constant of degradation of *Lunatic fringe* protein	0.37 *min*^−1^	16
82.	*k*_82_	Inhibition constant for Gsk3 inhibition of *Lunaticfringe* transcription induced by NICD	2.5 *min*^−1^	25
83.	*k*_83_	Hill coefficients for Notch oscillator	2.0	16
84.	*k*_84_	Scaling factor for Notch oscillator	0.3	16
85.	*k*_85_	Bimolecular rate constant for complex formation of NICD and Dsh	0.5 *min*^−1^	25
86.	*k*_86_	Bimolecular rate constant for degradation of complex of NICD and Dsh	9.0 *min*^−1^	25
