# Supplementary material for: Dynamical states, possibilities and propagation of stress signal
Source: Sci Rep. 2017 Jan 20;7:40596. doi: 10.1038/srep40596 (PMC5247771; doi:10.1038/srep40596)
Supplement: Supplementary Information [file srep40596-s1.pdf]

Supplementary Information to "Dynamical states, possibilities and propagation of stress signal"

Md. Zubbair Malik[1,2], Shahnawaz Ali[1,2], Soibam Shyamchand Singh[1,2], Romana Ishrat[1] and R.K. Brojen Singh[2*]
[1] *Centre for Interdisciplinary Research in Basic Sciences, Jamia Millia Islamia, New Delhi-110025, India.*
[2] *School of Computational and Integrative Sciences, Jawaharlal Nehru University, New Delhi-110067, India.*

I. DIFFERENT DYNAMICAL STATE

Recent advances in the microscopy have revealed the dynamical behaviour of many small signalling molecules in vitro [1–8]. In some of the conditions the dynamical properties have been shown to control differentiation in cell [5, 8, 9]. These examples suggest regulation of many unexplored pathways. In recent studies of Purvis et. al., 2012 [10], the normal and first damped oscillation state (Table 1 main article) corresponds to the pulsating stage as achieved by them after exposing the cells to gamma and UV radiation; where they also achieved the sustained oscillation state (3rd stage in our stage) that corresponded with the complete cell cycle arrest (see Fig 1. in Purvis et. al. 2012) [10]. Thus, pulsating stage (in our case stage 1 and 2) with increase in stress throws the cell into either DNA repair or the cell cycle arrest (stage 3). The dose given to the cell in terms of Nutilin-3, gave the stability to the cellular p53 concentration and thus, provided time for the synthesis of AIPs (anti apoptotic proteins) in the cell [11]. While on the other hand the increased level of (sustained oscillation stage 3) p53 by nutilin-3a was associated with the increased p21 concentration that promotes cell cycle arrest along with simultaneous shooting the level of caspases-6 and 7 (apoptotic) proteins [12, 13]. Thus, the increase in the level of caspases-6 and 7 promote the arrival of apoptosis (in our case stage 5) [13]. The stage 4 corresponds to the intermediate stage (stage 4) in between arrest of the cell cycle with increasing toxicity and apoptosis, that clearly captures the time lap for the synthesis of apoptotic proteins in p53 dependent manner [10, 12, 13].

II. METHOD

A. Model Description

Notch is the single pass and trans-membrane protein present in metazoan species, interaction between Notch-Delta proteins at the cell surface and on the same cell gave rise to the cis-inhibition of Notch [14]. Canonical notch signalling is initiated on the binding of DSL (Delta/Serrate/LAG-2) ligands on opposite cells (SuppFig1). Delta has also a second role of inhibiting notch activity when inside the same cell (Cis-inhibition). In case of trans-inhibition the endocytosis of Notch ligand complex is mediated by ADAM metalloprotease after cleaving Notch at S2. Now this fragment is cleaved by gamma-secretase at S3 to release NICD, which then is transported to the nucleus [15].

---

*Electronic address: brojen@jnu.ac.in (Corresponding author)

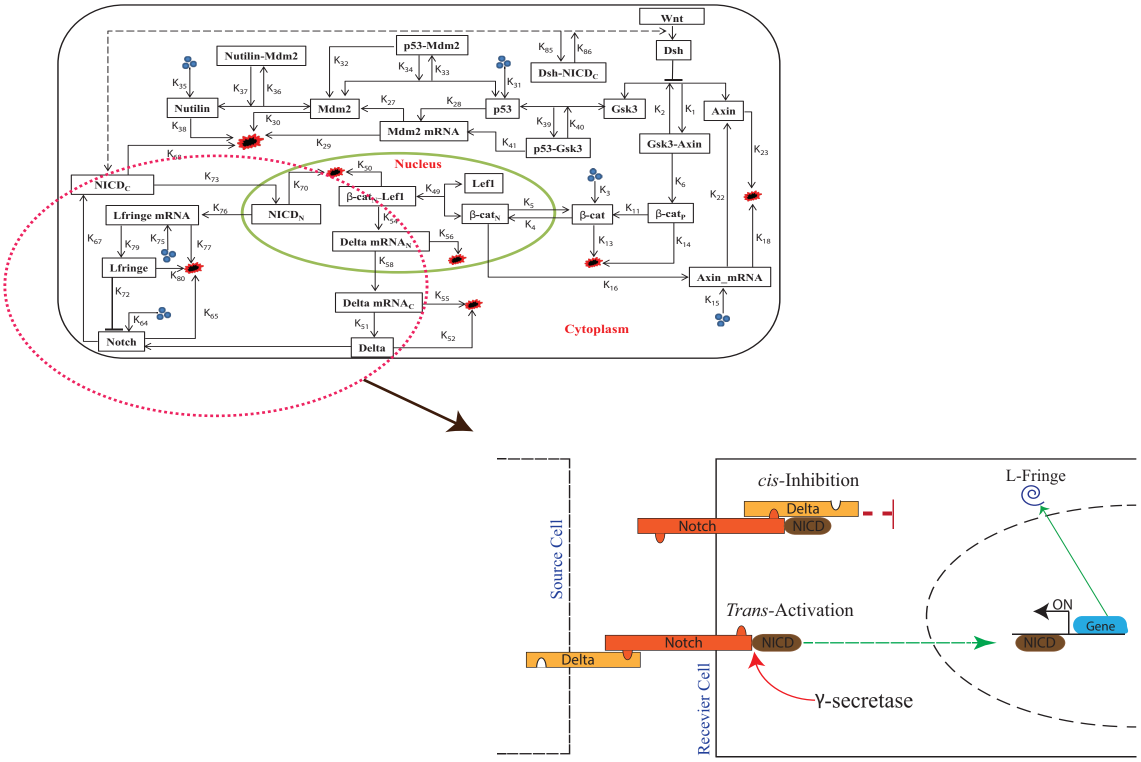

FIG. 1: Interaction of Cis-inhibition and Trans-activation in $Notch - Wnt - p53$ cross-talk model.

[1] Hoffmann, R., Levchenko, A., Scott, M. L. & Baltimore,D. The IB-NF-B signaling module: temporal control and selective gene activation. *Science*, **298**(5596), 1241-1245 (2002).

[2] Lahav, G., Rosenfeld, N., Sigal, A., Geva-Zatorsky, N., Levine, A.J., Elowitz, M.B., Alon, U. Dynamics of the p53-Mdm2 feedback loop in individual cells. *Nature genetics*, **36**(2), 147-150 (2004).

[3] Mettetal, J.T., Muzzey, D., Gmez-Uribe, C. and van Oudenaarden, A. The frequency dependence of osmo-adaptation in Saccharomyces cerevisiae. *Science*, **319**(5862), 482-484 (2008).

[4] Nelson, D.E., Ihekwaba, A.E.C., Elliott, M., Johnson, J.R., Gibney, C.A., Foreman, B.E., Nelson, G., See, V., Horton, C.A., Spiller, D.G. & Edwards, S.W. Oscillations in NF-B signaling control the dynamics of gene expression. *Science*, **306**(5696), 704-708 (2004).

[5] Suel, G.M., Garcia-Ojalvo, J., Liberman, L.M. & Elowitz, M.B. An excitable gene regulatory circuit induces transient cellular differentiation. *Nature*, **440**(7083), 545-550 (2006).

[6] Tay, S., Hughey, J.J., Lee, T.K., Lipniacki, T., Quake, S.R. & Covert, M.W. Single-cell NF-[kgr] B dynamics reveal digital activation and analogue information processing. *Nature*, **466**(7303), 267-271 (2010).

[7] Lee, T.K., Denny, E.M., Sanghvi, J.C., Gaston, J.E., Maynard, N.D., Hughey, J.J. and Covert, M.W. A noisy paracrine signal determines the cellular NF-B response to lipopolysaccharide. *Science signaling*, **2**(93), ra65 (2009)

[8] Santos, S.D., Verveer, P.J. & Bastiaens, P.I. Growth factor-induced MAPK network topology shapes Erk response determining PC-12 cell fate. *Nature cell biology*, **9**(3), 324-330, (2007).

[9] Hayward, P., Kalmar, T. & Arias, A. M. Wnt/Notch signals and information processing during development.*Development*,**135**(3), 411-424 (2008).

[10] Purvis, J. E., Karhohs, K. W., Mock, C., Batchelor, E., Loewer, A. & Lahav, G. p53 dynamics control cell fate.*Science*,**336**(6087), 1440-1444 (2008).

[11] Shin, S., Sung, B. J., Cho, Y. S., Kim, H. J., Ha, N. C., Hwang, J. I. & Oh, B. H. An anti-apoptotic protein human survivin is a direct inhibitor of caspase-3 and-7. *Biochemistry*, **40**(4), 1117-1123 (2001).

[12] Carter, B. Z., Mak, D. H., Schober, W. D., Koller, E., Pinilla, C., Vassilev, L. T. & Andreeff, M. Simultaneous activation of p53 and inhibition of XIAP enhance the activation of apoptosis signaling pathways in AML.*Blood*,**115**(2), 306-314 (2010).

[13] Zhang, Y., Fujita, N. & Tsuruo, T. Caspase-mediated cleavage of p21 Waf1/Cip1 converts cancer cells from growth arrest to undergoing apoptosis.*Oncogene*,**18**(5), (1999).

[14] Sprinzak, D., Lakhanpal, A., LeBon, L., Santat, L.A., Fontes, M.E., Anderson, G.A., Garcia-Ojalvo, J. & Elowitz, M.B. Cis-interactions between Notch and Delta generate mutually exclusive signalling states. *Nature*, **465**(7294), 86-90 (2010).

[15] Chapman, G., Major, J. A., Iyer, K., James, A. C., Pursglove, S. E., Moreau, J. L. M., & Dunwoodie, S. L. Notch1 endocytosis is induced by ligand and is required for signal transduction. *Biochimica et Biophysica Acta (BBA)-Molecular Cell Research*, **1863**(1), 166-177 (2016).
